# Opioid-Associated Amnestic Syndrome

**DOI:** 10.7759/cureus.20056

**Published:** 2021-11-30

**Authors:** Macey L Walker, Keshav Patel, Tong Li, Mahmoud Kassir

**Affiliations:** 1 Internal Medicine, Western Michigan University Homer Stryker M.D. School of Medicine, Kalamazoo, USA; 2 Internal Medicine, University of Illinois College of Medicine, Chicago, USA; 3 Family and Community Medicine, Western Michigan University Homer Stryker M.D. School of Medicine, Kalamazoo, USA

**Keywords:** opioid-associated amnestic syndrome, hippocampal edema, substance abuse, amnesia, opioid crisis

## Abstract

Opioid-associated amnestic syndrome (OAS) is a relatively new condition that is associated with opioid abuse and has increased in prevalence since the notable rise in opioid-related deaths and opioid-related hospitalizations of the opioid crisis. Patients often present with acute anterograde amnesia and current opioid abuse, most commonly fentanyl. OAS is frequently diagnosed when other potentially infectious or metabolic conditions such as encephalitis and seizures are ruled out, as these conditions can also present similarly to OAS. This case highlights the signs, symptoms, and hallmark characteristics of OAS, including bilateral hippocampal edema and anterograde amnesia.

## Introduction

A systematic review in 2020 identified only 40 cases of opioid-associated amnestic syndrome (OAS) [1]. While OAS is commonly associated with fentanyl use, it can also be seen with other opioids such as hydromorphone [2,3]. OAS may result from the effects of any of the following conditions: reversible vasospasm from vascular smooth muscle, vasculitis secondary to immune-mediated response, embolic effects from drug contaminations, and hippocampal infarction caused by neuronal hypermetabolism due to opioid overdose [4,5]. As of 2021, no formal diagnostic criteria for OAS exist. However, Barash JA et al. proposed potential diagnostic criteria for OAS to include new-onset amnesia greater than 24 hours, positive opioid toxicology, and bilateral hippocampal injury [1]. As identified by this systematic review, most cases of OAS were seen in males under the age of 40. In the United States during the year 2020, 1.6 million people were suffering from an opioid abuse disorder and over 70,000 people died of a drug overdose in 2019 [6]. The opioid crisis continues to be a major public health problem, and physicians should be aware of OAS.

## Case presentation

A 33-year-old male with a past medical history of polysubstance abuse presented to the ED after overdosing on heroin of an unknown dosage. He had overdosed earlier in the day on heroin but became responsive after his girlfriend administered naloxone. He subsequently overdosed again, but this time responded incompletely to a second naloxone dose prompting emergency medical services to transport him to the ED. His girlfriend reported that he had been having memory problems for the last few days, which involved him repeatedly asking her the same questions about tasks he intended on completing, but no other changes to his behavior. He reported current chronic heroin use via snorting, smoking one pack of cigarettes per day for an unknown period of time, occasional marijuana use, previous methamphetamine use, and denied any alcohol use.

**Figure 1:**
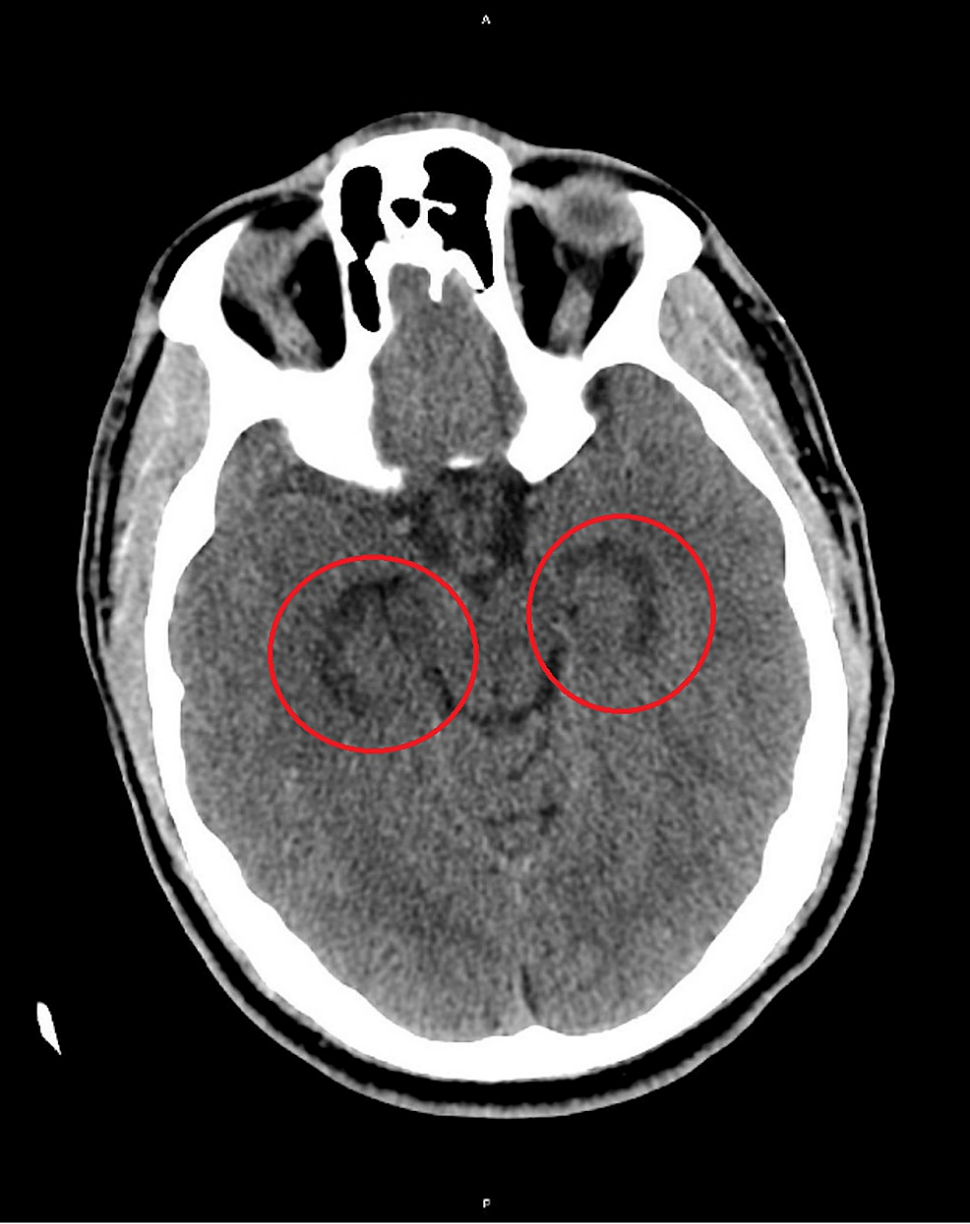
Symmetrical edema in bilateral hippocampi (red circles) in an otherwise unremarkable CT head (axial view) without contrast.

**Figure 2:**
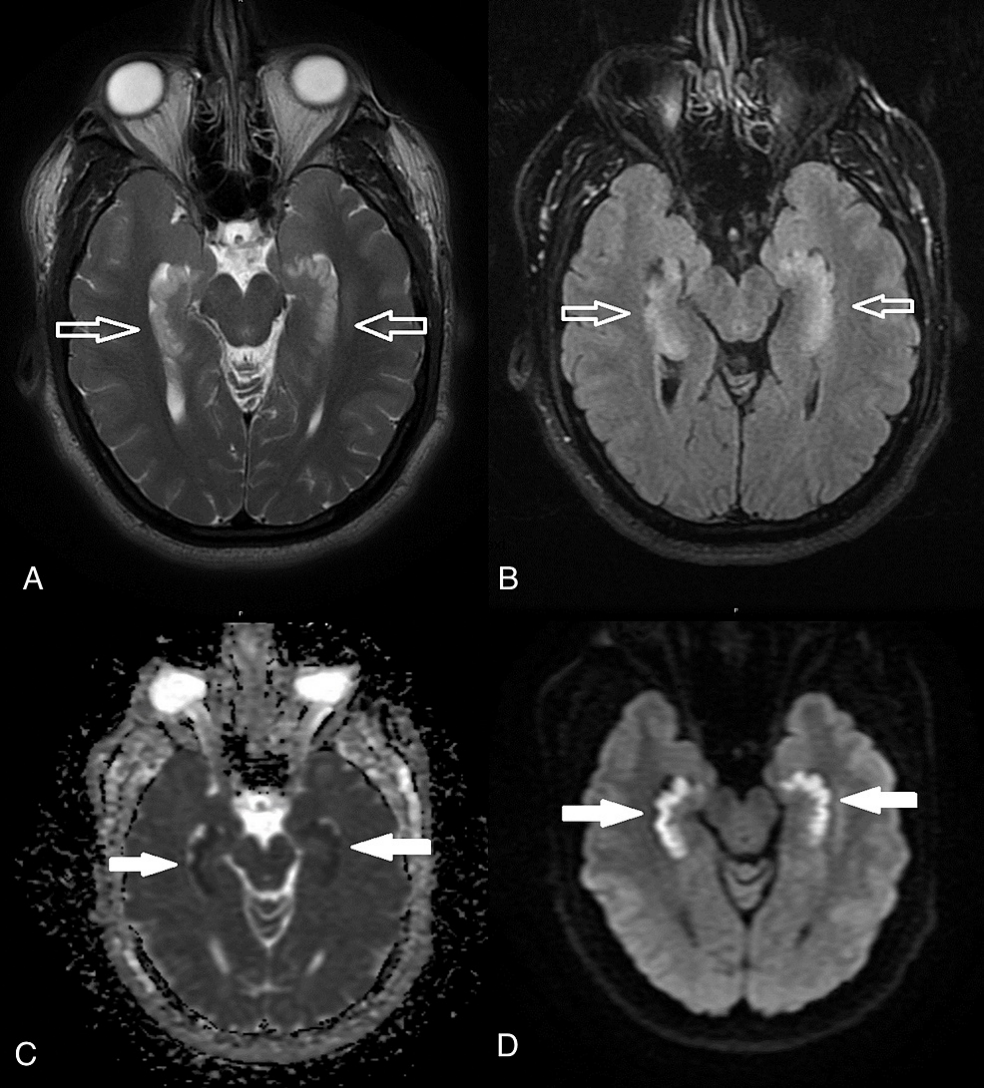
Axial views; T2-weighted (A), and FLAIR (B) demonstrate hyperintensity in the bilateral hippocampi region (white hollow arrows). On ADC (C), there is decreased signal intensity in the same hippocampi region (white solid arrows) along with corresponding increased signal intensity in DWI (D) which are suggestive of a diffusion restriction. ADC: Apparent diffusion coefficient; DWI: Diffusion-weighted imaging.

Initial vital signs were unremarkable. Physical exam was notable for short-term anterograde memory deficits. He was unable to follow and remember his plan of care and would frequently ask medical staff members what was going on and why things like imaging were being done, despite having asked these same questions just minutes before. The patient was awake and alert, but not oriented to date or location. He was noted to have a left frontotemporal scalp hematoma from a fall from two days prior, and mild right forearm weakness and tingling. Initial labs including complete blood count (CBC) and basic metabolic panel (BMP) were unremarkable. Lab results for herpes simplex virus (HSV), HIV, urine drug screen (UDS), and blood ethanol were all negative. CT and MRI of the brain revealed symmetric bilateral hippocampal edema (Figures 1-2). The patient was admitted for cerebral edema, altered mental status, and acute anterograde amnesia.

Initially, our differential diagnoses included toxic encephalitis, herpes encephalitis, or other viral encephalitis. Neurology was consulted and potential etiologies included infectious encephalitis for his amnesia and cerebral edema, and his right forearm weakness was from compression monotherapies.

IV thiamine was administered with no improvements in amnesia. A lumbar puncture was negative with no cells or bacteria in cerebrospinal fluid and the meningoencephalitis panel was negative. Blood cultures, echocardiogram, and EEG were to be considered if the condition worsened, but the patient’s conditions remained stable throughout his four-day hospital stay. On discharge, it was evident that the patient most likely had OAS since the presentation, imaging, and laboratory results had ruled out other causes, including infection, seizure, hypoglycemia, ischemia, and other metabolic conditions. Our patient’s medical imaging results, which include bilateral hippocampal edema that is exemplary of OAS, can be demonstrated in Figures 1-2. The patient's amnesia did not resolve or improve at the time of discharge and follow-up after discharge did not occur.

## Discussion

The initial negative UDS screen is a very interesting finding in our patient. The most likely cause for a negative UDS despite a clear clinical history would be an opioid substance that is not detected on routine UDS. Currently, with the opioid epidemic, there has been a surge in overdose involving synthetic opioid compounds [6]. Overdose deaths involving synthetic opioids other than methadone (mostly fentanyl) jumped from 9,580 in 2015, to 19,413 in 2016, and 36,359 in 2019 [7]. Such compounds will not show up on a routine UDS. In addition, many heroin users often unknowingly ingest synthetic opioid compounds. We believe our patient inadvertently consumed a synthetic opioid compound which led to his overdose. Therefore, clinicians cannot use a negative UDS to rule out opioid use as there are several synthetic opioids that will not be detected on UDS. 

CT and MRI of the brain revealed symmetric bilateral hippocampal edema which can be caused by conditions that result in hypoxemia, hypoxemia-ischemia, or seizures. Bilateral hippocampal edema and restricted diffusion have been documented to be frequently associated with anterograde amnesia and are typical of OAS [8].

Differential diagnoses for OAS include encephalitis, ischemia, seizures, hypoglycemia, and other metabolic conditions. Due to the novelty and low incidence of OAS, the long-term effects are not entirely clear. Some reports indicate that amnesic symptoms completely resolved for patients months after OAS diagnosis, while for other patients, severe symptoms persisted [9]. OAS is a rare diagnosis and patients with OAS may be misdiagnosed, especially when patients are using opioids that are undetectable on UDS. Additionally, the patient could either not disclose opioid use intentionally or due to amnestic symptoms.

## Conclusions

With synthetic opioid overdoses on the rise and considering that fentanyl is the opioid most frequently associated with OAS, this case report highlights that a negative UDS cannot rule out opioid use since several synthetic compounds may not be detected. Diagnosis of OAS should rely more on identifying the characteristic signs and symptoms for OAS, namely anterograde amnesia and bilateral hippocampal edema, and ruling out potential differential diagnoses. At this time, more research is needed to determine if it is always possible for amnestic symptoms to resolve and if there are therapeutic methods that could be shown to aid in this process.
